# Nasolabial cyst: presentation of a clinical case with CT and MR images

**DOI:** 10.1016/S1808-8694(15)30585-1

**Published:** 2015-10-19

**Authors:** Raphael Navarro Aquilino, Vitor José Bazzo, Reinaldo José Antônio Faria, Nayene Leocádia Manzutti Eid, Frab Norberto Bóscolo

**Affiliations:** 1 Master in dental radiology, Piracicaba Dentistry School (Faculdade de Odontologia de Piracicaba) - UNICAMP. Doctoral student in dental radiology, Piracicaba Dentistry School (Faculdade de Odontologia de Piracicaba) - UNICAMP. Professor of oral radiology, legal dentistry, deontology and bioethics. Professional orientation in the dentistry course, Gurupu Regional University (Universidade Regional de Gurupi) - UNIRG. Professor of the image diagnosis discipline in the medical course of the Gurupi Regional University (Universidade Regional de Gurupi) - UNIRG.; 2 Specialist in dental radiology, Bauru Dental School (Faculdade de Odontologia de Bauru) - USP. Master in deontology and legal dentistry - FOUSP. Doctoral student in dental radiology and image diagnosis, FOUSP. Professor of dental radiology, Presidente Prudente Dentistry School (Faculdade de Odontologia de Presidente Prudente) - UNOESTE.; 3 Specialist in prostheses and orthodontics - UNOESTE. Master's degree student in bucco-dental biology, Piracicaba Dental School (Faculdade de Odontologia de Piracicaba) - UNICAMP. Assistant professor of the dental materials discipline, Presidente Prudente Dentistry School (Faculdade de Odontologia de Presidente Prudente) - UNOESTE.; 4 Specialist in dental radiology, Craniofacial Anomalies Rehabilitation Hospital (Hospital de Reabilitação de Anomalias Craniofaciais) - USP. Master's degree student in dental radiology, Piracicaba Dentistry School (Faculdade de Odontologia de Piracicaba) - UNICAMP. Graduation student.; 5 Full professor of radiology, Piracicaba Dentistry School (Faculdade de Odontologia de Piracicaba) - UNICAMP. Gurupi Dentistry School (Faculdade de Odontologia de Gurupi) - UNIRG. Piracicaba Dentistry School (Faculdade de Odontologia de Piracicaba) - UNICAMP.

**Keywords:** nasolabial cyst, maxillary cysts, non-odontogenic cysts

## Abstract

The nasolabial cyst is an uncommon non-odontogenic cyst that develops in the lower region of the nasal ala; its pathogenesis is uncertain. This lesion grows slowly and measures between 1.5 and 3 cm; it is characterized clinically by a floating tumefaction in the nasolabial sulcus, which elevates the upper lip. The diagnosis is based on the clinical findings and, if necessary, image exams. This paper reports a case of a white 48-year-old Brazilian female patient that presented a firm tumor in the left ala of the nose; the clinical features suggested a nasolabial cyst. CT scans revealed an expanding tumor with soft tissue density located in the left ala of the nose. It measured 1.2 cm in diameter and had a clear and well-defined outline; its homogeneous density was about 50 HU. MR images revealed a circular lesion located in soft tissue; T1 and T2 weighted signals were hyperintense, as were images after fat suppression. The diagnosis was a nasolabial cyst, which was confirmed by histopathology after surgery.

## INTRODUCTION

Zuckerkandl originally described Nasolabial cysts in 1882; the first case itself was reported by McBride in 1892. In 1989, Brown-Kelly^1^ described this condition in greater detail.

In 1953, Klestadt^2^ investigated nasolabial cysts in depth, after which the lesion became named Klestadt's Cyst, in his honor. Since 1941, various other names had been used for this lesion, such as mucoid cyst, maxillary cyst, wind cyst, nasovestibular cyst, subalar cyst and nasoglobular cyst; Thoma^3^ suggested nasoalveolar cyst, but it was Rao^4^ (1951) who first used the term nasolabial cyst, defining it as a lesion located between the soft tissues of the upper lip and nasal vestibule. A cyst that erodes the surface of the maxilla is named nasoalveolar cyst.

There is still much debate about the origin of nasolabial cysts. It is thought that its pathogenesis is related to a period between the 4th and 8th weeks of intra-uterine life; at this time the maxillary process of the second brachial arch forms the base of the nose and the nasal alae. Midline fusion between each maxillary lateral palatine process and the base of the septum forms the hard palate, at the same time initiating formation of the nasal fossa. Aberrant changes at any of these fusion points may give rise to a fissure cyst.^5^ In 1953, Klestadt^2^ suggested that these cysts derived from ectodermal epithelial remains of embryological facial clefts along the fusion line between the middle and lateral nasal processes and the maxillary process; for this reason they would also be named fissure cysts.^6^, ^7^, ^8^ The other theory for explaining the genesis of these cysts is that they develop from cell remains derived from the lower opening of the nasolacrimal duct.^9^, ^10^, ^11^ Pseudostratified columnar epithelium lines this duct, and is similar to that often found on the walls of nasolabial cysts.^7^, ^12^

Signs and symptoms may include local pain, nasal block, and infection, which may cause tumefaction with significant inflammation and infection. This is clinically characterized by a floating mass in the nasolabial sulcus area that involves the nasal alae, extending to the ventralinferior portion of the pyriform margin, causing elevation of the lower lip.^13^ These cysts are usually unilateral (90%); they are bilateral in 10% of cases. They are more common in black females in the 4th and 5th decades of life.^11^, ^13^, ^14^

Cysts may cause bulging on the lateral wall of the nasal fossa floor, which becomes evident on occlusal maxillary radiographs.^15^ Osteolytic lesions, when present, may eventually involve the maxillary sinus.^16^

The differential diagnosis is made with odontogenic lesions such as follicular, periodontal and residual cysts, and neoplasms;^14^ only one case of carcinoma progressing from a nasolabial cyst has been described.10 Infected nasolabial cysts may be mistaken for FURÚNCULO of the nasal vestibule floor; except for this entity, however, the features of infected nasolabial cysts are very specific, and there is little doubt in the diagnosis.

Brown-Kelly first described the histopathology of this lesion in 1898.^17^ The cyst consists of respiratory epithelium (pseudostratified ciliated cylindrical or stratified ciliated cylindrical epithelium with goblet cells), although squamous metaplasia may occur in infected cysts.^7^ Fluid contained within cysts is produced by goblet cells.

The diagnosis of nasolabial cysts is essentially clinical. Bidigital palpation reveals a fluctuating tumefaction between the floor of the nasal vestibule and the gingivolabial sulcus, which helps to confirm the diagnosis.^7^ Radiograms do not detect this soft tissue lesion except when it causes significant maxillary bone erosion. More sophisticated image diagnosis, such as computed tomography (CT) and magnetic resonance imaging (MRI), may reveal the cystic nature of these lesions in greater detail and reliability, their relation with the nasal alae and the maxillary bone, as well as bone involvement, which facilitate the diagnosis.

The purpose of this study was to report a nasolabial cyst case and to describe its features on CT and MRI exams.

## CASE REPORT

**Figure 1:**
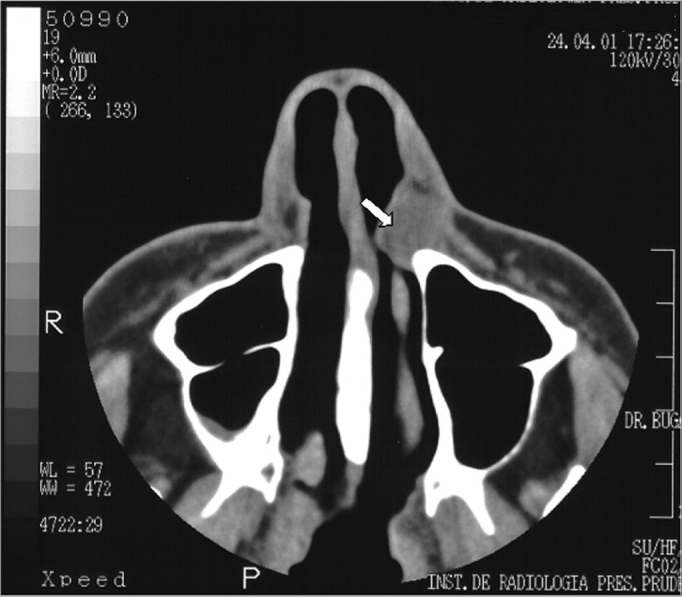
CT image (axial section) showing a heterogeneous mass suggesting soft tissue in the left nasal fossa (arrow).

A white woman aged 48 years presented for dental treatment at a private dental clinic and complained of a hard mass in the left nasal ala area that had developed over the past two years. The patient had previously sought a dermatologist, who raised the possibility of a tumor and referred her to an oncologist. The oncologist requested CT, which revealed a well-defined expansive mass with homogeneous density (around 50 Uh), similar to soft tissue, with 1.2 cm diameter, located in the left nose (Figure 1). Bone structures were preserved; there was slight thickening of the right maxillary sinus mucosa, and adequate transparency of the remaining paranasal cavities. MRI showed a rounded mass in the left nasal area. The mass was hyperintense in weighted T1 and T2 sequences, with part of the lesion showing an isointense content; T1 fat-suppression images of these areas were hyperintense, suggesting elevated protein content within (Figure 2A, 2B e 2C). Other facial region structures had normal signal morphology and intensity. The hypothesis was a cyst with elevated protein or dense mucous content, which suggested a nasolabial cyst.

**Figure 2A:**
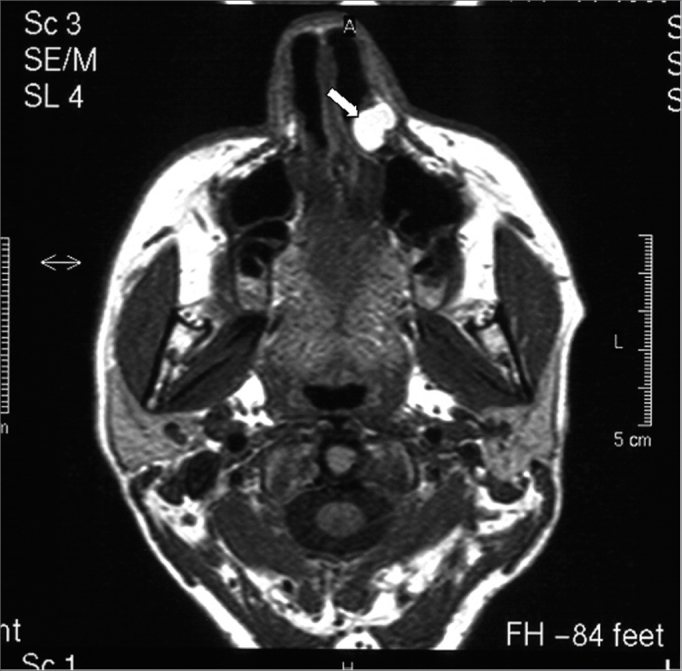
A-) T1-weighted MRI image (axial section). Note a hyperintense area in the left nose that suggests an elevated protein content.

**Figure 2B:**
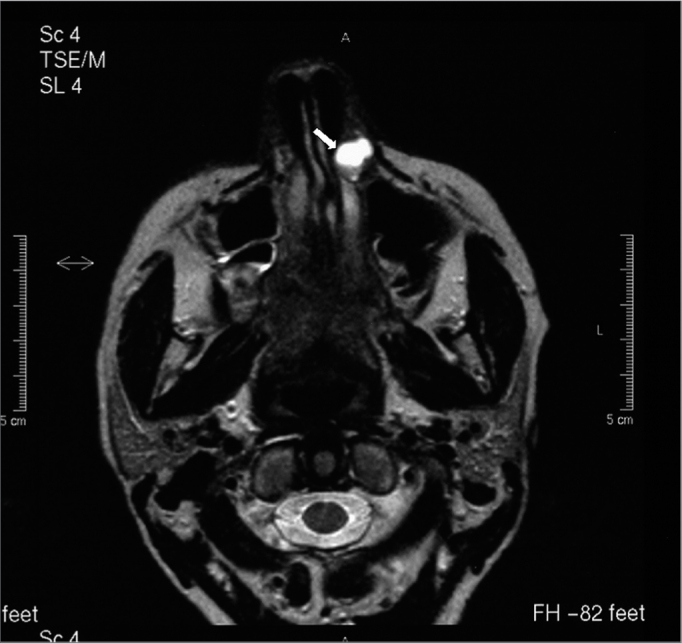
B-) T1-weighted MRI image (axial section). Note that the lesion contains isointense areas, and hyperintense areas in certain regions.

**Figure 2C:**
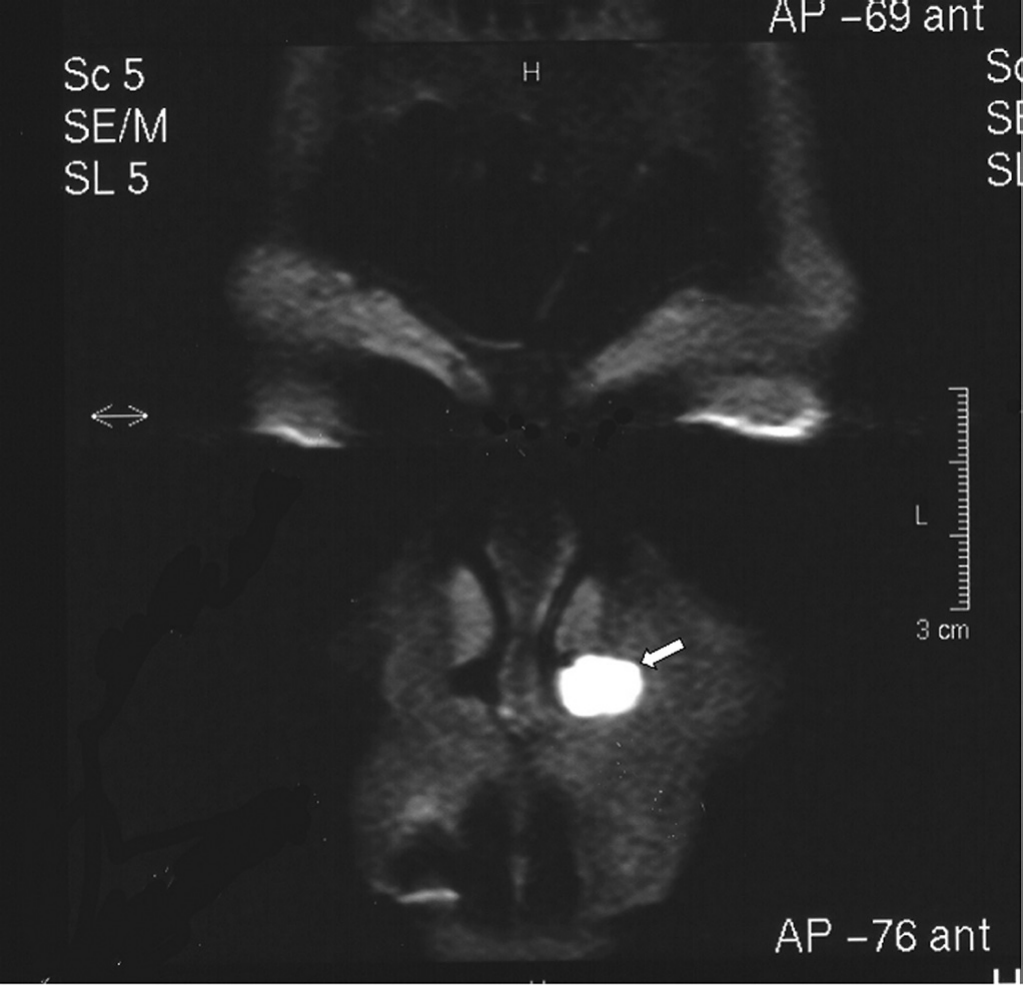
C-) Fat- suppressed MRI image (coronal section). Note hyperintense area in the lower left nasal fossa.

Other radiological exams were conventional dental radiographs (panoramic and periapical radiographs of teeth 12 and 22), which did not reveal significant changes that might suggest any bone lesion (Figures 3A and 3B). The physical examination did not show any typical sign of the lesion, such as elevation of the nasal alae and upper lip, or asymmetry. Upon palpation, there was a pain-free hard mass in the left nasal ala, which did not interfere with breathing.

**Figure 3A:**
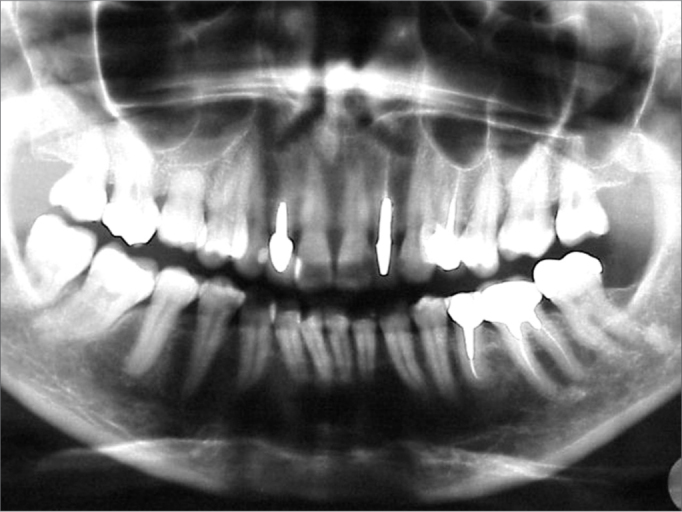
A-) Panoramic radiograph. This image shows no significant bone or dental alterations in the area of the lesion (note central and lateral incisor areas and upper left canines).

**Figure 3B:**
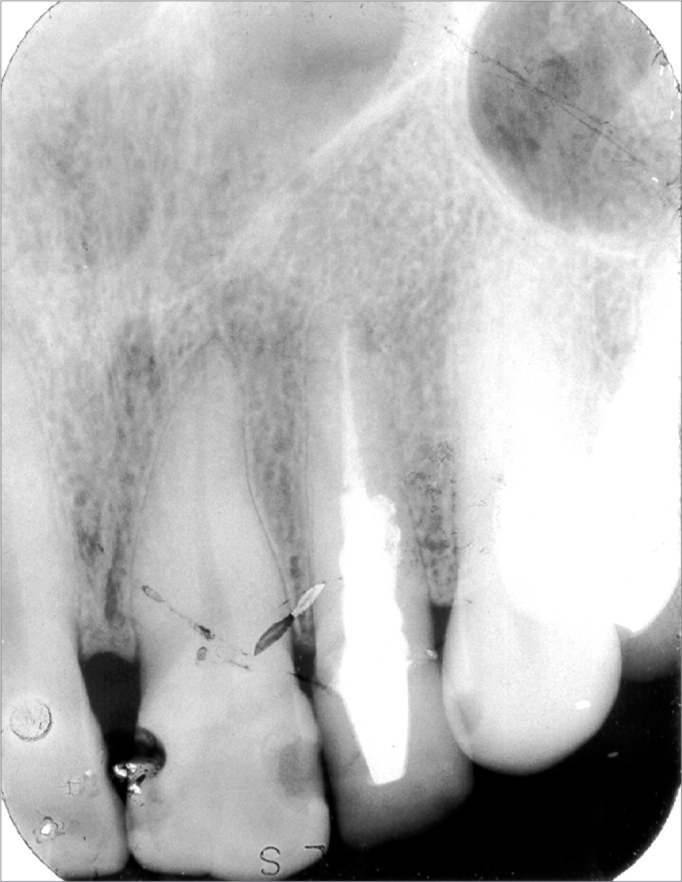
B-) Periapical radiograph of the central and upper left lateral incisors. This image shows no bone or dental alterations, which suggests a soft tissue lesion.

Therapy consisted of intra-oral surgical enucleation of the lesion under general anesthesia. An incision was made along the gingivolabial sulcus between the central incisor and the left first upper molar for removing the lesion. The cyst measured about 1.5 cm and contained a yellowish secretion. Histopathology revealed a fibrous cystic wall lined with pseudostratified cylindrical epithelium enclosing mucous secreting and goblet cells. The patient was followed-up postoperatively during five months. There were no postoperative symptoms, and the lesion did not recur within this period.

## DISCUSSION

This study presents a case of a nasolabial cyst in which the clinical history shows the difficulty of diagnosing a lesion that does not involve bone; such patients frequently seek many health professionals. David & O’ConnelL^18^ have stated that, although uncommon, nasolabial cysts are easily diagnosed and treated when health professionals know about the features of this condition. According to these authors, the rarity of this lesion in otorhinolaryngology is compounded by the fact that surgeons of the mouth treat many of them. In our opinion, the difficulty in diagnosing this lesion is their rarity.

Our case is not typical of the literature in terms of race; most of these lesions occur in black patients. Age and sex, however, were typical.^13^, ^14^ Van Bruggen et al.^19^ concluded that such lesions are bilateral in only 10% of cases: this percentage is 11.2% according to Roed-Peterson.^10^

The patient reported that the lesion developed within two years, and that there was no pain or discomfort and no esthetic effect. El-Hamd^14^ has reported, however, that some patients reported development of this lesion over three to five years, not having sought medical assistance due to its slow, asymptomatic growth and lack of pain or discomfort. Cohen & Hertzanu^20^ described the sudden growth to exuberance of a cyst within 2½ months after one year of slow development, in one of the nasolabial cyst cases.

El-Hamd^14^ reported that complications, when they occur, generally cause nasal block and a “cosmetic” appearance (redness) on the patient's face. Cohen & Hertzanu^21^ have stated that patients only seek therapy when there is deformity, nasal block of infection caused by this lesion.

Currently, there are modern methods that support physicians in making a diagnosis. The diagnosis of nasolabial cysts is almost exclusively clinical.^13^ We believe that, given the rarity of this condition, physicians use image methods to assure themselves about what type of lesion is present. Gomes et al.^13^ have suggested that CT provides a superior view of surrounding bone around the cyst; in the case of ultrasound, identifying and aspirating liquid from cysts is of little value, since the findings are uncharacteristic. Chinellato & Damante^15^ reviewed the histology of eight nasoalveolar cysts. According to these authors, cortical margins that refer to the anterior and lateral borders of these cysts are thin and convex in a lateral direction; when nasolabial cysts are present, there is expansion and sometimes thinning of this line, and convexity is inverted medially due to pressure from adjacent tissue, which may be seen on a total occlusal radiograph of the maxilla. Our findings are similar to these; we found slight bulging and thinning of the lateral wall of the nasal fossa. Shear^11^ published a study of nine patients showing that even a soft tissue cyst may at times erode the bone surface of the maxilla, which may be seen on radiographs.

Pruna et al.^21^ studied eight patients with nasal tumors in whom CT of the paranasal sinuses was done. These patients also underwent high frequency, gray-scale, and color Doppler ultrasound. Nasal hemangiomas were diagnosed in five patients; one patient had a submucosal gland cyst and one patient had a nasolabial cyst. These authors concluded that when a tumor in the anterior region of the nose is suspected, specific ultrasound with Doppler may help define the anatomical origin, local extension and the correct diagnosis in undefined cases; this might also be supported by other image methods and surgical procedures.

Pruna et al.^21^ have reported that nasal hemangiomas are seen on CT as a soft tissue mass that enhances significantly with contrast. These tumors may push the nasal septum medially and the nasal wall laterally, which is in agreement with our findings on CT images. On MRI, nasal hemangiomas show intermediate intensity in T1 and hyperintensity in T2; there is also hypointensity along the borders of these lesions. We found T1 and T2 hyperintensity in the tumor, as well as a hyperintense signal along the borders due to the high protein content of these cysts. Fat-suppressed T1-weighted images were also done, in which case the lesion was hyperintense.

Our findings differ from those of Curvé, Osguthorpe & Van Tassel;^22^ these authors presented MRI images of two nasolabial cyst cases that showed relative hyperintensity in T1-weighted images and isointensity in T2-weighted images.

The diagnosis in this study was based on clinical findings and image diagnosis, which described all of the findings of nasolabial cysts, and which were confirmed by histopathology.

## CONCLUSIONS

- CT and MRI are important exams for the diagnosis in suspected nasolabial cyst cases.
- MRI revealed cyst contents more clearly than CT.
- The diagnosis of nasolabial cysts should be based on clinical and imaging method findings.

## References

[1] Brown-Kelly A. (1898). Cyst of floor of nose. J Laryngol Rhinol Otol.

[2] Klestadt W (1953). Nasal cysts and the facial cleft cyst theory. Annals Otol Rhinol Laryngol.

[3] Thoma KH (1941). Nasoalveolar cysts. Am J Orthod.

[4] Rao RV (1995). Nasolabial cyst. J laryngol Otol.

[5] Robert CB (1970). Cyst lesions of the maxilla. Laryngoscope.

[6] Graamans K (1983). Nasolabial cysts: diagnosis mainly based on topography?. Rhinology.

[7] Kuriloff DB (1987). The nasolabial cyst-nasal hamartoma. Otolaryngol Head Neck Surg.

[8] Barzilai M (1994). Bilateral nasoalveolar cysts: case report. Clin Radiol.

[9] Bruggermann A (1930). Zysten als folgen von Entwicklungsstorungen im naseneingang. Arch Laryngol Rhinol.

[10] Roed-Petersen B (1969). Nasolabial cysts. Brit J Oral Surg.

[11] Shear GR (1985). Cysts of the jaws. J Oral Pathol.

[12] Waldrep AC, Capodanno JA (1966). Bilateral nasolabial cysts: report of case. J Oral Surg.

[13] Gomes CC (1992). Cistos Nasolabiais. Folha med.

[14] El-Hamd KEAA (1999). Nasolabial Cyst: a report of eight cases and a review of the literature. J Laryngol Otol.

[15] Chinellato LEM, Damante JH (1984). Contribution of radiographs in the diagnosis of nasoalveolar cyst. Oral Surg Oral Med Oral Pathol.

[16] Atterbury RA, Vazirani SJ, McNabb WJ (1961). Nasoalveolar cyst-oral surgery. Oral Med Oral Pathol.

[17] Burtschi TA, Stout RA (1963). Bilateral nasoalveolar cysts. Oral Surg.

[18] David VC, O’Connell JE (1986). Nasolabial cyst. Clin Otolaryngol.

[19] Van Bruggen AP (1985). Nasolabial cyst: a report of ten cases and a review of literature. J Dental Assoc South Africa.

[20] Cohen MA, Hertzanu Y (1985). Huge grow potential of nasolabial cyst. Oral Surg Oral Med Oral Pathol.

[21] Pruna X (2000). Value of sonography in the assessment of space-accupying of the anterior nasal fossa. J Clin Ultras.

[22] Cure JK, Osguthorpe JD, Van Tassel P (1996). AJNR Am J Neuroradiol.

